# Anatomical Variations of the Nasal Cavities and Paranasal Sinuses: A Systematic Review

**DOI:** 10.7759/cureus.12727

**Published:** 2021-01-15

**Authors:** Anna-Maria Papadopoulou, Dimosthenis Chrysikos, Alexandros Samolis, George Tsakotos, Theodore Troupis

**Affiliations:** 1 Anatomy, Medical School, National and Kapodistrian University of Athens, Athens, GRC; 2 Paediatrics, Penteli Children's Hospital, Athens, GRC

**Keywords:** paranasal sinus, anatomical variants, sinus surgery, ethnic

## Abstract

The anatomy of the nasal cavities and paranasal sinuses is one of the most varied in the human body. The aim of this study is to review the prevalence of anatomical variations in the sinonasal area. This systematic review was conducted according to the Preferred Reporting Items for Systematic Reviews and Meta-Analysis (PRISMA) guidelines. We performed on PubMed a literature search from October 2004 until May 2020. The search strategy included the following keywords: (‘paranasal sinus’ OR ‘frontal sinus’ OR ‘maxillary sinus’ AND (‘anatomical variants’ OR ‘anomalies’)). Fifty studies were eligible and included in the analysis. Overall, the studies encompassed a total of 18,118 patients included in this review. Most common anatomical variations include agger nasi cells, nasal septum deviation and concha bullosa. Other variations seen in this region are uncinate process variations, paradoxical middle turbinate, Haller, Onodi and supraorbital ethmoid cells, accessory ostia of maxillary sinus. Less common variations include any sinus aplasia, crista galli pneumatization and dehiscence of the optic or maxillary nerve, internal carotid artery and lamina papyracea. Anatomical variations of this region also differ among ethnic groups. This study highlights the amount, variability and significance of most anatomical variants reported in the literature in the last years. It is essential for the sinus surgeon to have a broad spectrum of knowledge not only of “the typical” anatomy but also all the possible anatomical variations. With modern imaging modalities, anatomical variations can be detected, and uneventful pitfalls might be prevented.

## Introduction and background

The anatomy of the nasal cavities and paranasal sinuses is probably one of the most frequently varied in the human body. Due to their complex three-dimensional structure and many morphological variations, understanding these anatomical aspects is of paramount importance to a sinus surgeon. Functional endoscopic sinus surgery (FESS) is one of the prime components in the management of chronic rhinosinusitis, as well as a number of other sinus diseases [1]. A computed tomography (CT) examination of the paranasal sinuses provides the anatomical “road map” to identify the presence of significant anatomical abnormalities with increased level of clarity and accuracy and should be studied thoroughly prior to any surgical approach [2,3].

Humans have four pairs of sinuses named by the bones of the skull that they pneumatize. The maxillary, ethmoid (divided into anterior and posterior cells) frontal and sphenoid sinuses, which are all lined by mucosa [2]. Normally, the frontal sinus drains into the frontal recess [4]. The maxillary sinus drains into the hiatus semilunaris, then into the middle meatus [5] and finally in the nasal cavity through the primary maxillary ostium [6]. The ostiomeatal complex is located in the region between the middle turbinate and the lateral nasal wall in the middle meatus and represents the region for drainage of anterior ethmoid, maxillary and frontal sinuses [7]. The spheno-ethmoidal recess, above and posterior to the superior concha, receives the opening of the sphenoid sinus, while the superior meatus receives the openings of the posterior ethmoidal cells [1-5].

The aim of this study is to review the prevalence of anatomical variations in sinonasal area. Detailed knowledge of possible variations is essential for the sinus surgeon to operate safely in this complex area that is separated from the orbit and cranial fossa by only the thinnest of bones [6,8].

## Review

Materials and methods

This systematic review was performed in accordance with the Preferred Reporting Items for Systematic Reviews and Meta-Analyses (PRISMA) guidelines. The protocol of this systematic review has been submitted to the Institutional Review Board of Department of Anatomy, National and Kapodistrian University of Athens, Greece, and is available upon request. Eligible articles were identified by a search of the Medline biblio­graphical database for the period from October 2004 up to May, 2020. The study protocol was agreed by all co-authors. The search strategy included the following keywords: (‘paranasal sinus’ OR ‘frontal sinus’ OR ‘maxillary sinus’ AND (‘anatomical variants’ OR ‘anomalies’)). Language restrictions were applied (only articles in English, French, and German were considered eligible); two investigators (AMP and DC), working independently, searched the literature and extracted data from each eligible study. Reviews were not eligible, while all prospective and retrospective studies, as well as case reports, were eligible for this systematic review. Manuscripts that did not state the names of the authors were excluded. In addition, we checked all the references of relevant reviews and eligible articles that our search retrieved, so as to identify potentially eligible conference abstracts. Titles of interest were further reviewed by abstract. Finally, reference lists of eligible studies were manually assessed in order to detect any potential relevant article (“snowball” procedure).

Results

Article Selection and Study Demographics

**Figure 1:**
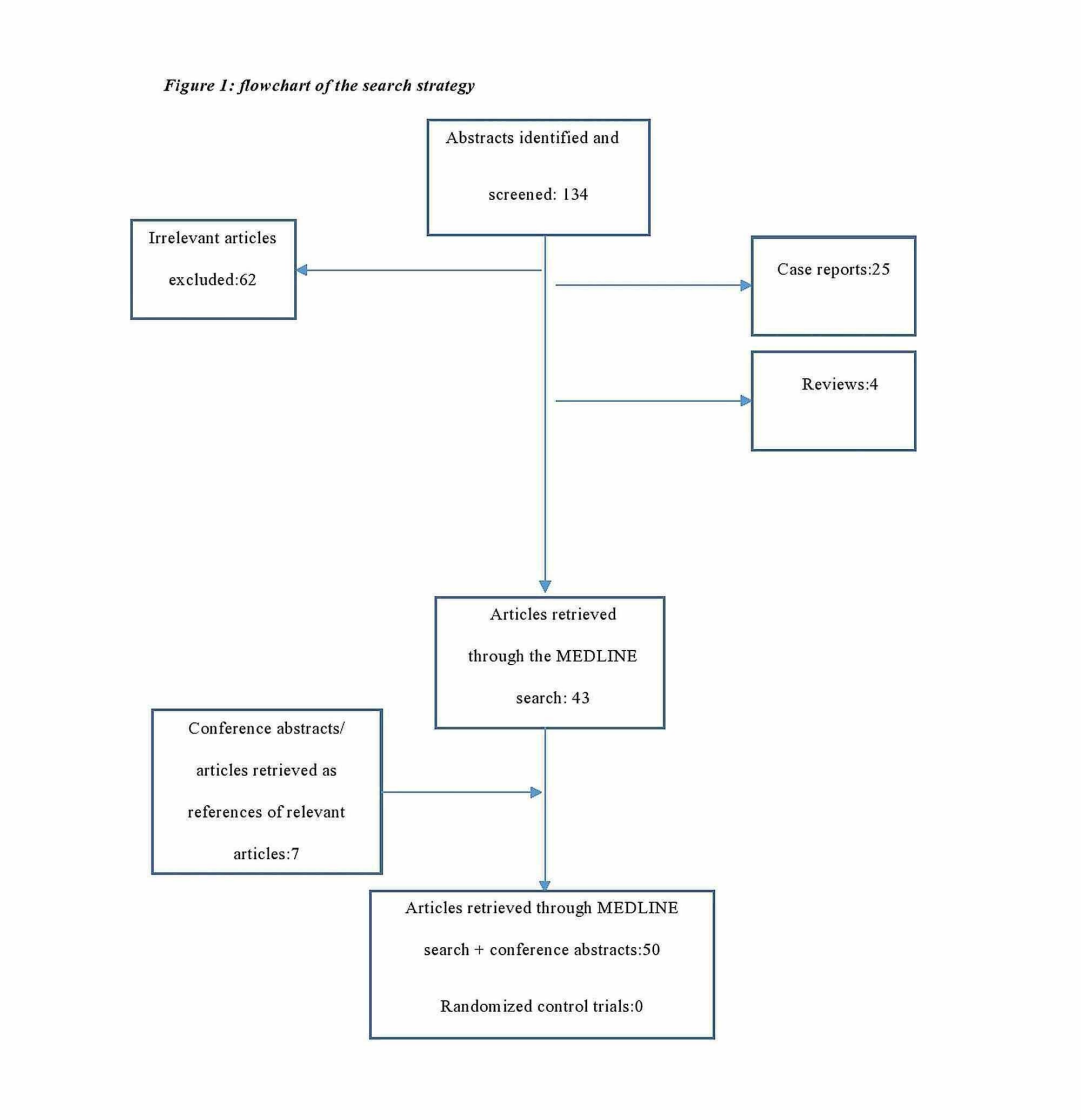
Flowchart of the search strategy

The search strategy retrieved 134 articles that were evaluated for full-text evaluation. Fifty studies were deemed eligible and were included in the analytic cohort. Overall, studies encompass a total of 18,118 patients that have been included in this systematic review. The search strategy is depicted in Figure 1.

Anatomical Variation Prevalence

There is an obvious variability in the prevalence of anatomical variations in the sinonasal region. Tables 1-6 summarize their prevalence according to the collected studies. Most common anatomical variations include agger nasi cells, nasal septum deviation and concha bullosa. Other variations seen in this region are uncinate process variations, paradoxical middle turbinate, Haller, Onodi and supraorbital ethmoid cells, accessory ostia of maxillary sinus. Less common variations include any sinus aplasia, crista galli pneumatization and dehiscence of the optic or maxillary nerve, internal carotid artery and lamina papyracea.

**Table 1:** Prevalence of anatomical variations in the sinonasal region AN: agger nasi, CB: concha bullosa, PMT: paradoxical middle turbinate, NSD: nasal septum deviation, HC: Haller cells, OC: Onodi cells, CG: pneumatized crista galli Empty cells correspond to not reported data.

First author, year	n	AN (%)	CB (%)	PMT (%)	NSD (%)	HC (%)	OC (%)	CG (%)
Mokhasanavisu, 2018 [1]	64	85	60	6.2		12.5	7.8	
Al-Abri, 2013 [2]	360	49	49	13	80	24	7.5	
Roman, 2015 [3]	130	93.2	35	8		25	8	
Choby, 2018 [4]	100	96.5						
Keast, 2008 [6]	180	86	28	32		29	21	
Nouarei, 2009 [7]	278		35			12		
Lien, 2010 [8]	192	89						
Kubota, 2015 [9]	150	88						
Turna, 2014 [10]	6224	18.3	57.2	9.1	59.1	14.8	13.5	9.4
Tiwari, 2015 [11]	85	7	76.4	10.5	88.2	3.5	1.6	
Kim, 2006 [12]	113	69	32.7	19.5	44.3	34.5	9.8	
Adeel, 2013 [13]	77		18.2	14.3	26	9.1	7.8	
Alsowey, 2017 [14]	240	60.6			57.5	61.3	55.8	
Fadda, 2012 [15]	200	17	34.5	4.5	41	16	6	
Kalairanasi, 2018 [16]	202		31.7					
Balikci, 2016 [17]	296		44.6		59.5			
Stallman, 2004 [18]	998		44		63.4			
Jain, 2012 [19]	77		46.7	41.5		28		
Yazici, 2019 [20]	120	92.5	44.2		36.6	20.8	30.8	
Cho, 2011 [21]	491		39.5	13.2				
Dasar, 2016 [22]	400	74.8	67.5	15.8	59.5	7.8	25.3	3.3
Shpilberg, 2015 [23]	192	83.3	26	15.6	98.4	39	12	9.9
Kasemsiri, 2011 [24]	187						49.5	
Shin, 2011 [25]	162						32.7	
Kaygusuz, 2013 [26]	99	61.6	45.4	13	72.7	16	8	22.2
Azila, 2011 [27]	240	81.2	44	17	58.3	56.6		
Leunig, 2008 [28]	641	80	22.2	0.9	73.6	16	8.4	14
Sommer, 2019 [29]	249	95.2						16.5
Rereddy, 2014 [30]	100	95	40	8	56	36	15	9
Kaplanoglu, 2013 [31]	500	63.8	30.4	14.6	81.8	15	10.6	

**Table 2:** Prevalence of anatomical variations in the sinonasal region SBC: suprabullar cells, EB: ethmoid bullae, LP: lamina papyracea, SOEC: supraorbital ethmoid cells Empty cells correspond to not reported data.

First author, year	n	Keros 1 (%)	Keros 2 (%)	Keros 3 (%)	SBC (%)	Enlarged EB (%)	Dehiscent LP (%)	SOEC (%)
Mokhasanavisu, 2018 [1]	64	68	32	0	44		6	
Al-Abri, 2013 [2]	360	30	34	36	13			
Choby, 2018 [4]	100				72			28.5
Keast, 2008 [6]	180	57	39	2				
Nouarei, 2009 [7]	278	92						
Lien, 2010 [8]	192				39.1			7.7
Kubota, 2015 [9]	150				37			6
Turna, 2014 [10]	6224							9.4
Tiwari, 2015 [11]	85					63.5		
Alsowey, 2017 [14]	240					56.3		
Fadda, 2012 [15]	200	11	5	1		23	0.5	
Dasar, 2016 [22]	400					6.3	0.3	9
Shpilberg, 2015 [23]	192					44.8	0.5	28
Kaygusuz, 2013 [26]	99					31.3		
Leunig, 2008 [28]	641	8	80	11.7	28.2			10.2
Sommer, 2019 [29]	249	9.2	82.3	8.4	88.8			9.2
Kaplanoglu, 2013 [31]	500							6.2
Comer, 2012 [32]	66							42.4

**Table 3:** Prevalence of anatomical variations in the sinonasal region Pn.: pneumatized, UP: uncinate process, ACP: anterior clinoid process Empty cells correspond to not reported data.

First author, year	n	Pn. UP (%)	Medialized UP (%)	Lateralized UP (%)	Atelectatic UP (%)	Pn. ACP (%)
Mokhasanavisu, 2018 [1]	64	3	9	1		
Roman, 2015 [3]	130	5				
Keast, 2008 [6]	180	2				
Nouarei, 2009 [7]	278					18
Turna, 2014 [10]	6224	7				20
Adeel, 2013 [13]	77	5.2				
Fadda, 2012 [15]	200	2	16	15	9	
Jain, 2012 [19]	77			5		
Yacizi, 2019 [20]	120					29.2
Dasar, 2016 [22]	400	13.8			0.5	25.5
Shpilberg, 2015 [23]	192					16.7
Kaygusuz, 2013 [26]	99	6				
Azila, 2011 [27]	240	2.5				
Leunig, 2008 [28]	641	8.8				16.5
Rereddy, 2014 [30]	100	11				
Kaplanoglu, 2013 [31]	500	3.8				23

**Table 4:** Prevalence of anatomical variations in the sinonasal region IFSP: inter-frontal septal cells, FC: frontal cells, FS: frontal sinus, FBC: frontal bullae cell Empty cells correspond to not reported data.

First author, year	n	IFSC (%)	FC1 (%)	FC2 (%)	FC3 (%)	FC4 (%)	FS aplasia (%)	FBC (%)
Mokhasanavisu, 2018 [1]	64	6	39	9	3	0.5		
Choby, 2018 [4]	100	30						
Lien, 2010 [8]	192	9.6	21.5	10.5	7.7	0		6.3
Kubota, 2015 [9]	150	8.6	37	6.3	4.3	1.3		7
Turna, 2014 [10]	6224						1.7	
Fadda, 2012 [15]	200		5	3	0.5			
Dasar, 2016 [22]	400	3.5					7.2	24.5
Leunig, 2008 [28]	641	11.9	17	6.8	12.5	0.1		16
Sommer, 2019 [29]	249	27.7						66
Tuncyurek, 2012 [33]	520		15.7	3.8	12.1	0.4		
Langille, 2012 [34]	399		26	6.4	2.1	0		
Wang, 2012 [35]	200	45						
Gotlib, 2015 [36]	305						5.7	
Aslier, 2016 [37]	74						4.1	
Nikolova, 2017 [38]	137						5	
Cakur, 2011 [39]	410						1.9	
Danesh-sani, 2011 [40]	565						6.9	
Yazici, 2019 [41]	120						33.3	

**Table 5:** Prevalence of anatomical variations in the sinonasal region SS: sphenoid sinus, ICA: internal carotid artery, ON: optic nerve Empty cells correspond to not reported data.

First author, year	n	ICA protrusion into SS (%)	ON protrusion into SS (%)	SS hypoplasia (%)
Mokhasanavisu, 2018 [1]	64	2	7	
Turna, 2014 [10]	6224	3	5.5	0.9
Dasar, 2016 [22]	400	46	35.3	14.5
Rereddy, 2014 [30]	100	17	18	
Yazici, 2019 [41]	120	20.8		5.8

**Table 6:** Prevalence of anatomical variations in the sinonasal region MS: maxillary sinus Empty cells correspond to not reported data.

First author, year	n	Accessory maxillary ostium (%)	MS hypoplasia (%)	MS septum (%)
Keast, 2008 [6]	150		7	
Hung, 2019 [5]	160	47.2		
Turna, 2014 [10]	6224		2.4	
Fadda, 2012 [15]	200		4	7
Jain, 2012 [19]	77	32		
Dasar, 2016 [22]	400	21.8	8	5.3
Kaplanoglu, 2013 [31]	500	16.4	0.8	12.4
Yazici, 2019 [41]	120		5	
Amine, 2020 [42]	300		5	34.6
Selcuk, 2008 [43]	330		4.6	22.8
Yenigun, 2016 [44]	377	19.1		
Bani-ata, 2020 [45]	928	29.5		

Ethnic Variation of Sinonasal Anatomy

Table 7 summarizes the anatomical variations of sinonasal anatomy among different ethnic groups. Badia et al. found statistically significant difference in the occurrence of concha bullosa, paradoxical middle turbinate, Haller and Onodi cells between Caucasian and Chinese patients [46]. Mokhasanavisou et al. compared the paranasal anatomy between Indians and Northeast Asians and found that Indians had a statistically higher sphenoid sinus volume, but among common and uncommon anatomical variants, there was no statistically significant difference between the two groups. They also showed type 1 Keros to be more common, which is contradicting to other studies [1]. According to Lien et al., the frequency of frontal recess cells in Taiwanese subjects is similar to that found in other Asian adult groups such as Chinese and Korean populations, while the prevalence of agger nasi and frontal bullar cells was closer to that in the Caucasian population [8]. Kubota et al. found that the prevalence of type 1 frontal cells in Japanese patients was almost as high as in Caucasians, but the prevalence of other frontal cells, especially type 2, was in line with findings in other Asian populations [9]. Keast et al. found no significant difference in the prevalence of anatomical variations between Polynesian and New Zealand Europeans [6]. Supraorbital ethmoid cells (SOEC) have been found to be more common in Caucasians, whereas suprabullar cells are more common in east Asian populations (Korean, Taiwanese, Chinese, Japanese) [4,9]. The different frequency of these variations among races can be attributed to genetic and environmental factors [9].

**Table 7:** Anatomic variations among different ethnic groups I: Indians, T: Taiwanese, J: Japanese, P: Polynesians, E: Europeans, Tur: Turkish, Pak: Pakistani, Ch: Chinese, Cau: Caucasians, O: Omani, AN: agger nasi, CB: concha bullosa, PMT: paradoxical middle turbinate, NSD: nasal septum deviation, HC: Haller cells, OC: Onodi cells, Pn. UP: pneumatized uncinate process, SBC: suprabullar cells, SOEC: supraorbital ethmoid cells, IFSP: inter-frontal septal cells, FC: frontal cells, FBC: frontal bullae cell, MS: maxillary sinus Empty cells correspond to not reported data.

Parameter (%)	I [1]	T [8]	J [9]	P [6]	E [6]	Tur [31]	Pak [13]	Ch [46]	Cau [46]	O [2]
AN	85	89	88	96	84	63.8		50	50	49
CB	60			61	60	30.4	18.2	8.5	21.5	45
NSD						81.8	26			
PMT	6.2			28	33	14.6	14.3	6.5	16	13
Pn. UP	3			9	2	3.8	5.2			
HC	12.5			33	28	15	9.1	5	12.5	24
OC	7.8			11	24	10.6	7.8	11	25	8
SBC	44	39.1	37							13
SOEC		7.7	6			6.2				
FBC		6.3	7							
IFSC	6	9.6	8.6							
FC1	39	21.5	37							
FC2	9	10.5	6.3							
FC3	3	7.7	4.3							
FC4	0.5	0	1.3							
Keros 1	68			54	58					30
Keros 2	32			44	38					34
Keros 3	0			0	0					36
MS hypoplasia				6	6	0.8				

Discussion

Interest in the anatomy of the paranasal regions was sparked by improvements in functional endoscopic surgery and CT [47]. A key concept indulged in endoscopic sinus surgery is that inflammatory sinus disease occurs due to impaired/deranged mucociliary drainage pathways of the sinuses into the ostiomeatal complex. FESS is aimed to restore this functional drainage [1]. Detailed knowledge of anatomic variations in the paranasal sinus region is critical for surgeons performing endoscopic sinus surgery as well as for radiologists involved in the preoperative work-up. Endoscopic examination in conjunction with CT has proven to be ideal combination in recent years and are already accepted as the “standard of care” for sinus diseases [11].

Nasal Septum Variations

Nasal septum deviation is the most common physical disorder regarding the nasal septum. The origin of the deviation has been described as either developmental (usually smooth, “C-shaped” or “S-shaped” nasal septum with occurrence more often in the anterior septum) or traumatic (usually irregular, angulated, and sometimes dislocated) [10,12]. It may be cartilaginous, osseocartilaginous or osseous [11]. Septal deviation can displace the middle turbinate, narrowing the middle meatus, making surgical access difficult and causing obstruction of the normal mucous flow and, consequently, secondary inflammation and infection [10,17]. The reported prevalence of septal variations in the literature ranges between 26% and 97% due to varying morphological features and the extent of deviation [14].

Nasal septal spurs may cause impaired nasal breathing and complicate surgical access by narrowing the nasal cavities, depending on their size [10].

Air cells of the nasal septum are commonly found within the posterior portion of the septum and communicate with the sphenoid sinus, allowing infections to spread to these cells. They are usually created due to the extension of air from the sphenoid sinus or crista galli and are not significant, but sometimes they may block the drainage of the middle meatus [10,13].

Turbinate Variations

Concha bullosa is the pneumatization of the concha and is one of the most common variations of the sinonasal anatomy. Middle turbinate pneumatization is most frequently encountered, while the superior turbinate is pneumatized rarely. As far as the inferior turbinate is concerned, only a few papers describe inferior concha bullosa and most of these have appeared as case reports [10]. Concha bullosa is a result of pneumatization of the osseous palate due to ethmoidal extension. It can be unilateral or bilateral, while its size varies [10]. It becomes apparent after seven years of age and continues its development even after the period of adolescence [15,16]. Its reported prevalence ranges from 15% to 80% and the highest prevalence is seen in patients with chronic sinusitis [14]. It has been classified based on the location as lamellar, bulbous and extensive concha bullosa. If the pneumatization is extensive, a large concha bullosa may cause significant problems by its size alone, such as headaches [15] with accompanying mucosal contact and/or a marked nasal obstruction. Bulbous and extensive types may require surgical correction. The concha bullosa, rarely, when filled with fluid and pus, results in mucopyocele, which must be differentiated from other nasal masses [16]. 

Many authors have found a strong relationship between the presence of a concha (unilateral or a dominant concha) and deviation of the nasal septal convexity away from the concha [17-19]. However, there is almost always maintenance of the nasal air channel between the medial aspect of the concha and the adjacent surface of the nasal septum. This implies that the deviation of the septum away from the concha is not the result of the concha pushing away the septum. Rather, there appears to be some as yet unknown developmental relationship between the concha and nasal septum. No information could be found suggesting whether the concha develops first and the nasal septum somehow “senses” the mass effect of the concha and correspondingly develops away from this side, or if the septal deviation develops first and then the concha enlarges to partially fill the expanded air channel. In either case, the septal deviation is often so great that there is compromise of the contralateral nasal air channels [18]. Apart from concha bullosa, Yazici et al. concluded that Onodi cells and anterior clinoid process pneumatization were more common in patients with nasal septal deviation [20].

Normally, the convexity of the middle concha is directed medially, towards the nasal septum. When paradoxically curved, the convexity is directed laterally, toward the lateral sinus wall [21]. It can be presumed as an etiologic factor for headache [24] and sinus disease because of the deformity and obstruction or alteration of nasal passage air flow dynamics, especially when associated with other variations; it has been associated mostly with septal deviation [10,14]. 

Ethmoid Cells Variations

The term “agger nasi” comes from the latin word for nasal mound. Agger nasi cells are considered the most anterior of all ethmoid cells, located in the anterior superior portion of the middle turbinate [10]. They are generally located bilaterally [22] and can pneumatize posteriorly to narrow the frontal recess, causing frontal sinusitis. Coronal and sagittal reformatted CT images are most helpful in identifying the agger nasi cells [10]. Their reported prevalence ranges from 10% to 98% [5]. Possible explanation of the lower frequency in pediatric patients is that agger nasi cells continue to develop even after the complete development of the ethmoid sinus under the effect of frontal sinus expansion. Importantly, these cells can provide access to frontal sinus during endoscopy [46].

Haller cells or the infraorbital cells, initially described by Albert von Haller in 1765, are the ethmoid cells that develop into the floor of orbit (i.e., the roof of maxillary sinus) adjacent to and above the maxillary sinus ostium [46]. If enlarged, they can significantly constrict the posterior aspect of the ethmoidal infundibulum and ostium of the maxillary sinus above. It is controversial that the Haller cells originate from anterior or posterior ethmoid cells. Their prevalence is remarkably variable, ranging from 8% to 57%, which could be attributed to the inconsistency in definition. It is a clinically significant variation because it has been implicated as a possible etiologic factor in recurrent maxillary sinusitis due to their negative influence on maxillary sinus ventilation by narrowing the infundibulum and ostium [10]. Additionally, the presence of Haller cells can increase the risk of orbital injuries during ethmoidectomy [23].

Onodi cell or sphenoethmoid air cell is a posterior ethmoid cell that is pneumatized far laterally and to some degree superiorly to the sphenoid sinus and is intimately associated with the optic nerve. The presence of an Onodi cell may possibly contribute to an increased risk of injury to the optic nerve and the internal carotid artery. That is why the identification of Onodi cells before surgery may be extremely valuable in decreasing the risk of such complication [10,24]. Usually, these cells interfere with the exposure of the edge of the sellar floor and should be removed in order to inspect and remove completely lesions of the sellar, parasellar and suprasellar compartments, achieving good results in pituitary surgery [25]. Infection in Onodi cells could also press on optic nerve and lead to retro-orbital pain [16]. Its frequency is reported to have a wide range, 2%-50%, which can be attributed to different definition criteria and difficulty of cell evaluation on the coronal plane CT [26].

SOEC are anterior ethmoid cells that extend superiorly and laterally over the orbit from the frontal recess. These cells represent pneumatization of the orbital plate on the frontal bone posterior to the frontal recess and the frontal sinus. They typically drain into the lateral aspect of the frontal recess. Up to 15% of adults may have one or more SOEC, with approximately 5% of frontal sinuses having multiple SOEC. Preoperative identification is essential because these cells can be readily mistaken for the frontal ostium during endoscopic dissection. Moreover, they may mimic the appearance of a septated frontal sinus or may give the appearance of multiple frontal sinuses [10]. On the other hand, Comer et al. found a significant association between the presence of frontal sinus septation and the presence of SOEC [32]. Their presence has been associated with orbital proptosis and can increase the risk of orbital damage during endoscopic sinus surgery. Failure to recognize a supraorbital cell with the anterior ethmoidal artery as a landmark during surgery increases the risk of skull base injury because cerebrospinal fluid (CSF) leaks and retraction of a lacerated anterior ethmoidal artery into the orbit can occur [27].

Bulla ethmoidalis is the largest and most nonvariant air cell in the anterior ethmoid complex. It is formed by pneumatization of the bulla lamella, or second ethmoid basal lamella, and it is like a bleb on the lamina papyracea. Hyperpneumatized ethmoid bulla is located between middle concha and uncinate process and could displace it towards medial [22].

Uncinate Process Variations

The uncinate process is a key bony structure in the lateral nasal wall. Together with the adjacent ethmoid bulla, it defines the hiatus semilunaris that forms an outlet for a recess, the infundibulum, which is directed anteriorly and inferiorly. The maxillary sinuses open into the posterior aspect of the infundibulum via the ostium; during FESS, the first procedure to display maxillary sinus is uncinectomy [23]. When the free margin of uncinated process is enlarged or deformed, it can compress the infundibulum, producing impaired sinus ventilation. Other, less important variations include pneumatization (2%-14%) and hypoplasia [14]. Although the exact mechanism by which uncinate pneumatization occurs is not known, it has been proposed that it is due to the growth of agger nasi cells into the most anterosuperior region of the uncinate process. This variation has been implicated in narrowing of the infundibulum, producing impaired sinus ventilation [27]. Notably, lateral displacement and hypoplasia of the uncinate process have been associated with maxillary sinus hypoplasia [6]. When the uncinate process inserts into the lamina papyracea, the ethmoid infundibulum is closed superiorly to form a blind pouch called the terminal recess [8].

Frontal Recess Cells

Cells superior to agger nasi are called front-ethmoidal (Kuhn) cells [22]. Bent and Kuhn described four distinct types of frontal sinus cells (FSC) in 1994. Type 1 FSC is the most frequent and describes a single cell in the frontal outflow tract above the agger nasi. Type 2 cells exist as a tier of cells above the agger nasi. A type 3 cell extends cephalad past the frontal recess into the frontal sinus itself. Most studies identify type 2 cells as the second most common, with some exceptions [28,33,34]. A type 4 FSC was initially described as a cell that existed entirely within the frontal sinus, but more recently it has been defined as a cell that extends >50% of the height of the frontal sinus and is quite rare. Alternatively, the International Frontal Sinus Anatomy Classification (IFAC) provides a precise nomenclature for cells in the frontal recess, classifying cells based on their anatomical origin [4,29]. There are several other frontal recess cells, which include frontal bullar cells, suprabullar cells and inter-frontal sinus septal cells [35]. Before frontal sinus surgery, the variable frontal recess cells in each patient must be analyzed to plan a strategy for dissecting all cells disturbing the nasofrontal recess [9]. Moreover, Eviatar et al. described a newly observed frontal sinus anatomical variant, the front-septal rostrum in 30.5% of patients [48].

Frontal Sinus Variations

Knowledge of the frontal sinus anatomy is important for surgeons performing endoscopic sinus surgery, frontal sinus balloon sinuplasty, craniotomy or external approaches [36]. The frontal sinuses are essentially the only paranasal sinuses that are absent at birth, because, on average, these sinuses do not reach up into the frontal bone until the age of about six years. Because the left and right frontal sinuses develop independently, a significant asymmetry between these sinuses can arise in the same individual. An absence of pneumatization in the frontal bone results in frontal sinus aplasia. Although frontal sinus aplasia is not rare in the literature, its frequency is variable between different populations. More specifically, the frequency of bilateral absence of the frontal sinus has been reported in 2% to 33%, whereas the incidence of a unilateral absence has been reported to be between 0.8% and 7.4% [37-39]. In addition, bilateral frontal sinus aplasia is more common among female subjects [39,47]. Besides, climatic conditions, local inflammations and the mechanical stress of mastication can affect the size of frontal sinus [40,47].

Maxillary Sinus Variations

The maxillary sinus septum divides the maxillary antrum into bony compartments and is the most frequently encountered maxillary sinus variation in the literature, with a mean prevalence of 29% [42]. The primary septa are assumed to be formed during the embryonic development of the midline of the face. Secondary septa, on the other hand, are assumed to be formed secondary to bone resorption in the sinus base due to alveolar ridge atrophy following tooth loss in the process of maxillary sinus pneumatization. Selcuk et al. revealed that the position of antral septa was frequently vertical at anterior and horizontal at posterior and found a significant correlation between the anteriorly localized maxillary sinus septa and infraorbital fissure enlargement. They are thought to strengthen the sinus structure or mastication functions. It has also been reported in the literature that they may interfere with endoscopic sinus surgical procedures like extraction of foreign body, pathologic sinus mucosa or remnants of tooth roots. Moreover, irrigation and drainage of all compartments are to be done separately during surgery [43].

Maxillary sinus hypoplasia is an uncommon condition that may be misdiagnosed as chronic sinusitis [10]. Its prevalence has been reported to be 1%-11%. Maxillary sinus may become hypoplastic during its development in the embryologic period, accompanying syndromes such as Apert, Crouzon and Treacher Collins [49], or later due to traumatic, iatrogenic or structural causes. Despite the major opinion that infundibular obstruction may cause negative pressure leading to maxillary underdevelopment, there are opponents to this theory basing on the fact that maxillary sinus hypoplasia cases with totally normal ostiomeatal complex are encountered [43]. Maxillary sinus hypoplasia predisposes to lamina papyracea injuries and orbital penetration during endoscopic sinus surgery; therefore, this abnormality must be recognized as well as associated anatomical variations, especially prior to sinus surgery. It may also lead to dental problems by causing canine fossa elevation. It may clinically lead to silent sinus syndrome by causing hypoglobus and enophthalmos, as well. In addition, some patients may present to the ophthalmology department complaining of orbital asymmetry and double vision. Selcuk et al. also found significant correlation between maxillary sinus hypoplasia and orbital enlargement [43]. Maxillary sinus aplasia is a very rare congenital anomaly [9]. Ozcan et al. were the first to describe that frontal sinus hypoplasia/aplasia was more common in patients with hypoplastic maxillary sinuses. On the other hand, the maxillary sinus is over-pneumatized when the longest horizontal or vertical axis of the maxillary sinus is 90% or more of the relevant orbital size [49].

The widening of the posterior ethmoid cells into the maxillary sinus is known as ethmomaxillary sinus and it drains into the superior meatus. Its incidence is reported to be 0.7%-2% and is most frequently accompanied by a hypoplastic maxillary sinus. If not determined with CT prior to endoscopic sinus surgery, it may cause anatomical disorientation during the operative procedure [43].

Another anatomical variation that is not commonly found in anatomical texts is Sieur cells. These cells are identifiable between the maxillary sinus and pterygopalatine fossa, and Craiu et al. showed that they were present in 34%-42% of studied cases [47].

Accessory maxillary ostium may be present at varying degrees (20%-50%). It is located at 5-10 mm superior to the attachment point of the superior concha and it often opens to the lateral nasal wall and rarely to the infundibulum [44]. A possible mechanism for the development of accessory maxillary ostia is impediment of the main ostium by mucosal edema due to chronic sinusitis or other anatomical or pathological factors in the middle meatus that leads to rupture of membranous part of the nasal wall [45]. Its presence has been reported to cause re-entry of sinus secretions drained through the primary maxillary ostium, but it is still not clear whether it has a direct association with morphological changes of the sinus mucosa [5].

Finally, Lantos et al. have been the first to systematically describe the prevalence of infraorbital nerve protrusion into the maxillary sinus, revealing that it is not an uncommon variant (10.8%). The protrusion of this branch of the trigeminal nerve into the maxillary sinus, instead of normally traversing the orbital floor, can lead to an increased risk of injury during endoscopic sinus surgery or open surgical approaches, such as the Caldwell-Luc procedure [50].

Sphenoid Sinus Variations

The sphenoid sinus and adjacent bony structures may show various degrees of pneumatization. Considerable variations including cavernous sinus, internal carotid artery, optic and vidian canals are intimately related to sphenoid sinuses. More extensively, the optic canal is the place where the optic nerve is the least nourished throughout its course; therefore, it is very susceptible to injury through direct inflammatory invasion of the sinus diseases, and additionally, there is a risk of blindness if the surgeon damages the nerve within the sinus [10]. The reported prevalence for optic nerve protrusion into the sphenoid sinus ranges from 7% to 35% [50]. When the anterior clinoid process is pneumatized, mostly by the sphenoid or ethmoid sinus or both [41], the optic nerve protrudes against the superior lateral sinus wall [10]. Dasar et al. found a statistically significant association between anterior clinoid process pneumatization and optic nerve protrusion into the sphenoid sinus [22].

Furthermore, a dehiscence in the bone covering internal carotid artery may lead to direct contact of the artery with sinus mucosa that may lead to infection occurring within the cavernous sinuses. On the other hand, if this variation has not been noticed by the surgeon preoperatively, carotid artery injury could result in blindness or fatal hemorrhage [10]. The prevalence of internal carotid artery dehiscence and protrusion varies widely from 2% to 23% and 5.2% to 67% respectively [40]. Rereddy et al. found a significant association between dehiscent or protruding optic nerve and dehiscent or protruding internal carotid artery [30].

Postsellar pneumatization from the sphenoid sinus, particularly pneumatization of the dorsum sella, may result in the penetration of the posterior wall of the sphenoid with resultant CSF leak during trans-sphenoidal pituitary surgery [23].

Insertion of an intersphenoid sinus septum onto the carotid canal has been reported in 4.7% [50]. Another anatomical variant is the sphenomaxillary plate, a condition where a posteriorly over-pneumatized maxillary sinus is united with the sphenoid sinus via a bony septum. This structure should be recognized on the CT images in order to avoid orbital complications during surgery [43].

Aplasia of the sphenoid sinuses, lastly, is an extremely rare phenomenon. The diagnosis of sphenoid sinus hypoplasia is potentially important in patients in whom trans-sphenoidal hypophysectomy is contemplated [10].

Olfactory Fossa Variations

Knowledge of height and width of the olfactory fossa allows understanding of the upper limit of dissection and gives confidence to the surgeon [6]. Keros classified the ethmoid roof into three groups, based on the depth of cribriform plate from the ethmoid roof. In Type I, the olfactory fossa is less than 3 mm deep; the ethmoid roof is almost in the same plane as the cribriform plate. For Type II, the olfactory fossa is 4 to 7 mm deep [6,31]. In the literature, the incidence of this type varies from 5% to 80%, but is mostly above 50% as the most frequent type [41]. In Type III, the olfactory fossa is greater than 8 mm deep and the ethmoid roof lies significantly above the cribriform plate. The lateral lamella is longest in Type III and this configuration presents the greatest risk of perforation into the anterior cranial fossa [6].

Other Variations

The crista galli sits in the midline above the cribriform plate. Embryologically, crista galli is derived from the ethmoid bone, and as such, it would seem reasonable that any eventual pneumatization of the crista galli would come from the ethmoid complex. However, there is a possibility that pneumatization of crista galli could also come from the adjacent frontal sinuses [10].

In the literature, pneumatization of the pterygoid process was reported to be between 25% and 57%. When pneumatization expands into the plates, the floor of the sinus shows a definite ridge that corresponds to the vidian canal [10]. In particular, Dasar et al. observed a very significant association between pterygoid process pneumatization and vidian nerve protrusion into the sphenoid sinus [22]. Significant associations were also found between the pneumatized pterygoid process and dehiscent or protruding optic nerve, dehiscent or protruding internal carotid artery, and anterior ethmoid arteries below the skull base, according to Rereddy et al. [30].

Another uncommon anatomical variant is dehiscence of the lamina papyracea, which can lead to the prolapse of orbital contents into the ethmoidal sinuses and puts the patient at risk of hemorrhage or damage to the orbit during endoscopic intranasal ethmoidectomy [23].

The results from the collected studies highlight the wide variability of the reported prevalence for most anatomical variations. There might be several reasons for this discrepancy. For example, most studies differ from each other due to study design and sample. Some studies report the prevalence of anatomical variants based on CT scans of patients with symptoms regarding the sinonasal area, such as chronic rhinosinusitis, while others were conducted on both symptomatic patients and healthy controls. Moreover, genetic and environmental factors contribute to the different frequency of anatomical variations among different ethnic groups, as mentioned above. Finally, not all authors use the same definitions, classification systems and radiological parameters for anatomical variations and this could also lead to the wide divergence of their reported prevalence.

## Conclusions

Surgical anatomy of the sinonasal area is complex but important for many different clinical and surgical applications. This study highlights the amount, variability and significance of most anatomical variants reported in the literature during the last years. When operating near so many vital structures, the best management of any potential complication is prevention. Hence, it is essential for the sinus surgeon to understand not only the “standard” anatomy but also all the possible variants described in this study. With modern imaging modalities, anatomical variations can be detected, and potential pitfalls can be anticipated.

## References

[1] Mokhasanavisu VJP, Singh R, Balakrishnan R, Kadavigere R (2019). Ethnic variation of sinonasal anatomy on CT scan and volumetric analysis. Indian J Otolaryngol Head Neck Surg.

[2] Al-Abri R, Bhargava D, Al-Bassam W, Al-Badaai Y, Sawhney S (2014). Clinically significant anatomical variants of the paranasal sinuses. Oman Med J.

[3] Roman RA, Hedeşiu M, Gersak M, Fidan F, Băciuţ G, Băciuţ M (2016). Assessing the prevalence of paranasal sinuses anatomical variants in patients with sinusitis using cone beam computer tomography. Clujul Med.

[4] Choby G, Thamboo A, Won TB, Kim J, Shih LC, Hwang PH (2018). Computed tomography analysis of frontal cell prevalence according to the International Frontal Sinus Anatomy Classification. Int Forum Allergy Rhinol.

[5] Hung K, Montalvao C, Yeung AWK, Li G, Bornstein MM (2020). Frequency, location, and morphology of accessory maxillary sinus ostia: a retrospective study using cone beam computed tomography (CBCT). Surg Radiol Anat.

[6] Keast A, Yelavich S, Dawes P, Lyons B (2008). Anatomical variations of the paranasal sinuses in Polynesian and New Zealand European computerized tomography scans. Otolaryngol Head Neck Surg.

[7] Nouraei SA, Elisay AR, Dimarco A, Abdi R, Majidi H, Madani SA, Andrews PJ (2009). Variations in paranasal sinus anatomy: implications for the pathophysiology of chronic rhinosinusitis and safety of endoscopic sinus surgery. J Otolaryngol Head Neck Surg.

[8] Lien CF, Weng HH, Chang YC, Lin YC, Wang WH (2010). Computed tomographic analysis of frontal recess anatomy and its effect on the development of frontal sinusitis. Laryngoscope.

[9] Kubota K, Takeno S, Hirakawa K (2015). Frontal recess anatomy in Japanese subjects and its effect on the development of frontal sinusitis: computed tomography analysis. J Otolaryngol Head Neck Surg.

[10] Turna O, Aybar M, Karagoz Y, Tuzcu G (2014). Anatomic variations of the paranasal sinus region: evaluation with multidetector CT. Istanbul Med J.

[11] Tiwari R, Goyal R (2015). Study of anatomical variations on CT in chronic sinusitis. Indian J Otolaryngol Head Neck Surg.

[12] Kim HJ, Jung Cho M, Lee JW, Tae Kim Y, Kahng H, Sung Kim H, Hahm KH (2006). The relationship between anatomic variations of paranasal sinuses and chronic sinusitis in children. Acta Otolaryngol.

[13] Adeel M, Rajput MS, Akhter S, Ikram M, Arain A, Khattak YJ (2013). Anatomical variations of nose and para-nasal sinuses; CT scan review. J Pak Med Assoc.

[14] Alsowey AM, Abdulmonaem G, Elsammak A, Fouad Y (2017). Diagnostic performance of multidetector computed tomography (MDCT) in diagnosis of sinus variations. Pol J Radiol.

[15] Fadda GL, Rosso S, Aversa S, Petrelli A, Ondolo C, Succo G (2012). Multiparametric statistical correlations between paranasal sinus anatomic variations and chronic rhinosinusitis. Acta Otorhinolaryngol Ital.

[16] Kalaiarasi R, Ramakrishnan V, Poyyamoli S (2018). Anatomical variations of the middle turbinate concha bullosa and its relationship with chronic sinusitis: a prospective radiologic study. Int Arch Otorhinolaryngol.

[17] Balikci HH, Gurdal MM, Celebi S, Ozbay I, Karakas M (2016). Relationships among concha bullosa, nasal septal deviation, and sinusitis: retrospective analysis of 296 cases. Ear Nose Throat J.

[18] Stallman JS, Lobo JN, Som PM (2004). The incidence of concha bullosa and its relationship to nasal septal deviation and paranasal sinus disease. Am J Neuroradiol.

[19] Jain R, Stow N, Douglas R (2013). Comparison of anatomical abnormalities in patients with limited and diffuse chronic rhinosinusitis. Int Forum Allergy Rhinol.

[20] Yazici D (2019). The analysis of computed tomography of paranasal sinuses in nasal septal deviation. J Craniofac Surg.

[21] Cho JH, Park MS, Chung YS, Hong SC, Kwon KH, Kim JK (2011). Do anatomic variations of the middle turbinate have an effect on nasal septal deviation or paranasal sinusitis?. Ann Otol Rhinol Laryngol.

[22] Dasar U, Gokce E (2016). Evaluation of variations in sinonasal region with computed tomography. World J Radiol.

[23] Shpilberg KA, Daniel SC, Doshi AH, Lawson W, Som PM (2015). CT of anatomic variants of the paranasal sinuses and nasal cavity: poor correlation with radiologically significant rhinosinusitis but importance in surgical planning. AJR Am J Roentgenol.

[24] Kasemsiri P, Thanaviratananich S, Puttharak W (2011). The prevalence and pattern of pneumatization of Onodi cell in Thai patients. J Med Assoc Thai.

[25] Shin JH, Kim SW, Hong YK (2011). The Onodi cell: an obstacle to sellar lesions with a transsphenoidal approach. Otolaryngol Head Neck Surg.

[26] Kaygusuz A, Haksever M, Akduman D, Aslan S, Sayar Z (2014). Sinonasal anatomical variations: their relationship with chronic rhinosinusitis and effect on the severity of disease-a computerized tomography assisted anatomical and clinical study. Indian J Otolaryngol Head Neck Surg.

[27] Azila A, Irfan M, Rohaizan Y, Shamim AK (2011). The prevalence of anatomical variations in osteomeatal unit in patients with chronic rhinosinusitis. Med J Malaysia.

[28] Leunig A, Betz CS, Sommer B, Sommer F (2008). Anatomic variations of the sinuses; multiplanar CT-analysis in 641 patients. (Article in German). Laryngorhinootologie.

[29] Sommer F, Hoffmann TK, Harter L, Döscher J, Kleiner S, Lindemann J, Leunig A (2019). Incidence of anatomical variations according to the International Frontal Sinus Anatomy Classification (IFAC) and their coincidence with radiological sings of opacification. Eur Arch Otorhinolaryngol.

[30] Rereddy SK, Johnson DM, Wise SK (2014). Markers of increased aeration in the paranasal sinuses and along the skull base: association between anatomic variants. Am J Rhinol Allergy.

[31] Kaplanoglu H, Kaplanoglu V, Dilli A, Toprak U, Hekimoğlu B (2013). An analysis of the anatomic variations of the paranasal sinuses and ethmoid roof using computed tomography. Eurasian J Med.

[32] Comer BT, Kincaid NW, Kountakis SE (2013). The association between supraorbital ethmoid air cells and orbital proptosis in patients with chronic rhinosinusitis. Int Forum Allergy Rhinol.

[33] Tuncyurek O, Songu M, Adibelli ZH, Onal K (2012). Frontal infundibular cells: pathway to the frontal sinus. Ear Nose Throat J.

[34] Langille M, Walters E, Dziegielewski PT, Kotylak T, Wright ED (2012). Frontal sinus cells: identification, prevalence, and association with frontal sinus mucosal thickening. Am J Rhinol Allergy.

[35] Wang M, Yuan F, Qi WW, Cheng JY, Yuan XP, Han L, Xing ZM (2012). Anatomy, classification of intersinus septal cell and its clinical significance in frontal sinus endoscopic surgery in Chinese subjects. Chin Med J.

[36] Gotlib T, Kuźmińska M, Held-Ziółkowska M, Osuch-Wójcikiewicz E, Niemczyk K (2015). Hidden unilateral aplasia of the frontal sinus: a radioanatomic study. Int Forum Allergy Rhinol.

[37] Yüksel Aslier NG, Karabay N, Zeybek G, Keskinoğlu P, Kiray A, Sütay S, Ecevit MC (2016). The classification of frontal sinus pneumatization patterns by CT-based volumetry. Surg Radiol Anat.

[38] Nikolova S, Toneva D, Georgiev I, Lazarov N (2018). Digital radiomorphometric analysis of the frontal sinus and assessment of the relation between persistent metopic suture and frontal sinus development. Am J Phys Anthropol.

[39] Çakur B, Sumbullu MA, Durna NB (2011). Aplasia and agenesis of the frontal sinus in Turkish individuals: a retrospective study using dental volumetric tomography. Int J Med Sci.

[40] Danesh-Sani SA, Bavandi R, Esmaili M (2011). Frontal sinus agenesis using computed tomography. J Craniofac Surg.

[41] Yazici D (2019). The effect of frontal sinus pneumatization on anatomic variants of paranasal sinuses. Eur Arch Otorhinolaryngol.

[42] Amine K, Slaoui S, Kanice FZ, Kissa J (2020). Evaluation of maxillary sinus anatomical variations and lesions: a retrospective analysis using cone beam computed tomography. J Stomatol Oral Maxillofac Surg.

[43] Selcuk A, Ozcan KM, Akdogan O, Bilal N, Dere H (2008). Variations of maxillary sinus and accompanying anatomical and pathological structures. J Craniofac Surg.

[44] Yenigun A, Fazliogullari Z, Gun C, Uysal II, Nayman A, Karabulut AK (2016). The effect of the presence of the accessory maxillary ostium on the maxillary sinus. Eur Arch Otorhinolaryngol.

[45] Bani-Ata M, Aleshawi A, Khatatbeh A, Al-Domaidat D, Alnussair B, Al-Shawaqfeh R, Allouh M (2020). Accessory maxillary ostia: prevalence of an anatomical variant and association with chronic sinusitis. Int J Gen Med.

[46] Badia L, Lund VJ, Wei W, Ho WK (2005). Ethnic variation in sinonasal anatomy on CT-scanning. Rhinology.

[47] Craiu C, Rusu MC, Hostiuc S, Săndulescu M, Derjac-Aramă AI (2017). Anatomic variation in the pterygopalatine angle of the maxillary sinus and the maxillary bulla. Anat Sci Int.

[48] Eviatar E, Golan Y, Gavriel H (2018). Fronto-septal rostrum: prevalence, classification and clinical implications. J Laryngol Otol.

[49] Ozcan KM, Hizli O, Sarisoy ZA, Ulusoy H, Yildirim G (2018). Coexistence of frontal sinus hypoplasia with maxillary sinus hypoplasia: a radiological study. Eur Arch Otorhinolaryngol.

[50] Lantos JE, Pearlman AN, Gupta A, Chazen JL, Zimmerman RD, Shatzkes DR, Phillips CD. (2016). Protrusion of the infraorbital nerve into the maxillary sinus on CT: prevalence, proposed grading method, and suggested clinical implications. AJNR Am J Neuroradiol.

